# Reducing greenhouse gas emissions via harvest residue management in eucalyptus afforestation on Brazilian sandy soils

**DOI:** 10.3389/fpls.2025.1633436

**Published:** 2025-08-11

**Authors:** Jackson Freitas Brilhante de São José, Bruno Britto Lisboa, Frederico Costa Beber Vieira, Josiléia Acordi Zanatta, Elias Frank Araujo, Juscilaine Gomes Martins, Andressa Classer Bender, Eduardo Carniel, Cimelio Bayer, Luciano Kayser Vargas

**Affiliations:** ^1^ Department of Agricultural Research and Diagnosis, Department of Agriculture, Livestock, Sustainable Production and Irrigation of Rio Grande do Sul, Porto Alegre, Brazil; ^2^ Universidade Federal do Pampa, São Gabriel, Brazil; ^3^ Embrapa Floretas, Colombo, Brazil; ^4^ CMPC, Celulose Riograndense, Guaíba, Brazil; ^5^ Brazilian Institute of Environment and Renewable Natural Resources, Porto Alegre, Brazil; ^6^ Department of Soil Science, Faculty of Agronomy, Universidade Federal do Rio Grande do Sul, Porto Alegre, Brazil

**Keywords:** reforestation, carbon stock, nitrous oxide, methane, soil quality

## Abstract

**Introduction:**

The greenhouse gas balance is a central theme in discussions related to forest ecosystems. In this context, the present study evaluated the impact of five eucalyptus harvest residue management systems on atmospheric C-CO_2_ retention in soil, greenhouse gas (GHG) emissions, and the global warming potential (GWP) in *Eucalyptus saligna* plantations.

**Methods:**

The management systems examined were: AR - all harvest residues retained on soil; NB - harvest residues kept on soil, except bark; NBr - harvest residues kept on soil, except branches; NR - all harvest residues (bark, branches, leaves) removed; NRs - all residues from the previous rotation and new plantation litter removed using shade cloth. Soil emissions of nitrous oxide (N_2_O) and methane (CH_4_) were monitored over 12 months (October 2016 to October 2017). Soil samples were collected to a depth of one meter to assess atmospheric C-CO_2_ retention.

**Results and discussion:**

Annual N_2_O emissions were low (0.11–0.23 kg N-N_2_O ha^−1^ year^−1^) and showed no clear relationship with the amount of nitrogen added through residues. The soil consistently functioned as a methane sink across all management systems, with CH_4_ fluxes ranging from –2.56 to –3.91 kg C-CH_4_ ha^−1^ year^−1^. The highest rate of C-CO_2_ retention in soil (–5,540 kg C-CO_2_ ha^−1^ year^−1^) was observed under the AR management system, while the lowest (–1,752 kg C-CO_2_ ha^−1^ year^−1^) occurred under the NRs system. AR management also resulted in the lowest global warming potential (–33,946 kg C-CO_2_ ha^−1^ year^−1^), primarily due to soil C-CO_2_ retention (15.43%) and carbon accumulation in biomass and wood products (84.57%). These findings demonstrate that retaining eucalyptus harvest residues in subtropical sandy soils, in conjunction with carbon sequestration in wood products, constitutes an effective forest management strategy for mitigating global warming.

## 1 Introduction

Forest ecosystems are recognized for their efficiency in fixing atmospheric CO_2_ and storing substantial amounts of carbon (Lal, 2005). They also play a pivotal role in the greenhouse gas (GHG) balance, generally acting as sources of CO_2_ and N_2_O while serving as sinks for CH_4_ (Walkiewicz et al., 2025). In this context, while forest degradation and deforestation are major contributors to the rise in atmospheric GHG concentrations (van der Werf et al., 2009; Reygadas et al., 2023), planted forests offer a potential mitigation strategy (Waring et al., 2020). Worldwide, planted forests occupy approximately 294 million hectares across the five continents (FAO, 2020). Of this total, eucalyptus plantations occupy approximately 25 million hectares in tropical and subtropical regions (Mao et al., 2024), with 7.8 million of this area located in Brazil (IBÁ, 2024).

In recent years, there has been a growing economic interest in using forest harvesting residues as a source of renewable energy (Udali et al., 2024). In Brazil, it is projected that roughly 6.4 million tons of wood residues are generated annually in the eucalyptus and pine harvesting processes (Pincelli et al., 2017). From this perspective, several forestry companies worldwide are adopting the whole-tree harvesting system, which collects, in addition to wood, other components such as branches, bark, and leaves to facilitate the removal of these materials from the field (Nieminen et al., 2016).

However, the removal of these residues in eucalyptus areas can have adverse effects on soil quality. Possible consequences include reduced soil fertility (Menegale et al., 2016), increased susceptibility to erosion (Wichert et al., 2018), negative influence on biological activity (Chaer and Tótola, 2007) and reduced soil organic C stocks (Rocha et al., 2018). Removing eucalyptus harvesting residues can be even more impacting in sandy soils, with drastic decreases in soil organic C stocks and soil C retention rates (Epron et al., 2015; São José et al., 2023).

A further aspect that should be considered and studied is the impact of removing eucalyptus harvest residues on GHG emissions. In crop areas, recent studies have evaluated the influence of residue management on N_2_O and CH_4_ emissions (Pitombo et al., 2017; Vasconcelos et al., 2018; Langeroodi et al., 2019; Mirzaei et al., 2024), and such studies have generally found that maintaining crop residues contributes to reducing emissions of these two gases. In forest areas, the information is scarcer.

The major source of N_2_O emissions in agriculture is the application of nitrogen fertilizers, but the N present in plant residues also contributes substantially to the emissions (Syakila and Kroeze, 2011). The magnitude of this contribution depends on the chemical composition of the residue added to the soil (Li et al., 2016). Residues with a low C/N ratio increase N_2_O emissions (Chen et al., 2013). In contrast, residues with a high C/N ratio favor nitrogen immobilization, resulting in lower emissions (Muhammad et al., 2011).

Forest soils are recognized as significant CH_4_ sinks due to the oxidation of this GHG by methanotrophic microorganisms (Wigley et al., 2024). However, factors such as soil temperature, moisture, fertilization, and residue management determine whether the soil will act as a source or sink of CH_4_ (Vasconcelos et al., 2018). Generally, the input of organic substrates under anaerobic conditions promotes methanogenesis, resulting in high CH_4_ emissions (Zhang et al., 2015). On the other hand, increased soil porosity facilitates the transport of CH_4_ to methanotrophs, enhancing its oxidation and leading to lower net emissions (Prajapati and Jacinthe, 2014).

The GHG emissions can be used to calculate the global warming potential (GWP) of different eucalyptus harvest residue management. The GWP compares the warming potential of each gas to that of CO_2_, which is taken as a reference (Bayer et al., 2016). Specifically, CH_4_ and N_2_O have a 100-year global warming potential 34 and 298 times higher than CO_2_, respectively (Zhou et al., 2023). Studies in subtropical regions have demonstrated the potential of reforestation to reduce GWP values (de Godoi et al., 2016; Martins et al., 2015); however, these studies did not consider the effects of eucalyptus harvest residue management on soil carbon stocks and GHG emissions. Therefore, this study aimed to evaluate the influence of eucalyptus harvest residue and litter management on the GHG balance in sandy soil in the Brazilian subtropics.

## 2 Materials and methods

### 2.1 Experimental area and treatments

The experimental area was in the city of Barra do Ribeiro, in Rio Grande do Sul, the southernmost state of Brazil. The site lies near the coordinates 30°23’S and 51°07’W, at an altitude of approximately 30 m above sea level. The local climate is classified as humid subtropical (Cfa) according to the Köppen classification, with an average annual precipitation of approximately 1400 mm and no distinct dry season. The highest average monthly temperature does not exceed 25 °C, while the lowest is around 14 °C, with occasional light frosts. The local soil is classified as Quartzipsamment, characterized by a sandy texture, weak structure, low water storage capacity, and low cation exchange capacity (**Supplementary Table S1**). More details about the experimental area can be found in São José et al. (2020; 2022; 2023). The experiment was established in 2010 using *Eucalyptus saligna* (clone 2864). Each plot measured 30 × 30 m and was planted with 100 trees arranged in a grid of 10 rows by 10 plants per row. For the analyses, we considered an inner subplot measuring 18 × 18 m, consisting of 6 rows by 6 plants. The experimental design was a completely randomized block with four replicates and five treatments. The treatments involved five different eucalyptus residue management practices, described as follows:

1. AR – All forest residues were left on the soil (i.e., bark, branches, leaves, and the litter layer from the previous rotation), with only the trunk wood removed.
2. NB – Same as AR, but the bark was also removed.
3. NBr – Same as AR, but branches were also removed.
4. NR – All eucalyptus residues (including bark, branches, leaves, and litter) were removed.
5. NRs – Same as NR, but a shade net was also used to prevent litter from the new plantation from reaching the soil surface.

### 2.2 C and N input by crop residues and litter

The input of C and N was assessed at the beginning of the experiment. Branches, bark, and leaves from the previous crop were collected, their mass quantified, ground, and analyzed for C and N content to estimate the amounts contributed by each component. The accumulation of litter up to the sixth year of the current cultivation, as well as the addition of C and N through residue management, were estimated as described by São José et al. (2023).

### 2.3 Soil organic C stocks

Disturbed and undisturbed soil samples were collected in July 2016, in the 6th year of cultivation, to determine organic C content and soil density, respectively. Samples were collected from the following soil layers: 0–2.5, 2.5–5, 5–10, 10–20, 20–30, 30–50, 50–75, and 75–100 cm. Carbon stocks were calculated for the 0–100 cm profile based on equivalent soil mass, using as a reference the system in which all harvest residues and litter from the current crop were removed (NRs). Annual rates of atmospheric C–CO_2_ retention in the soil (Mg ha^−1^ year^−1^) were calculated as the ratio between the difference in soil C stocks relative to the reference system (NRs) and the duration of cultivation, as shown in the equation:


$$\begin{array}{c} \text{C}-\text{CO}2\;\text{anual}\;\text{retention}\;\text{rate}\\ =\frac{\text{Treatment}\;\text{soil}\;\text{C}\;\text{stock}\;-\;\text{NRs}\;\text{C}\;\text{stock}}{6\;\text{years}} \end{array}$$

### 2.4 C accumulation in wood products

The accumulation of C in wood products (WPs) under different residue management treatments was estimated based on the forest productivity. In the 6th year of afforestation, the diameter at 1.30 meters height (DBH) was measured using a tape measure, and the total height (h) of the experimental trees was measured using a hypsometer. Forest productivity was estimated by the average annual increase (AAI, m^3^ ha^-1^ year^-1^), based on the volume obtained after six years using the volume equation with bark, using the model by Leite et al. (1995) presented below:


$$\begin{array}{c} V\;=\;0.000048\times DBH^{1.720483}\times h^{1.180736}\times e^{\left(-3.00555\right)\;\times \;\left(tx/DBH\right)\;}\\ \times \;\{1-{\left(\frac{d}{DBH}\right)}^{1+0.228531\;\times d}\}+\;Є \end{array}$$

where DBH represents the diameter at 1.3 meters height; h the total height; tx equals to 0, for volume with shell, or 1, for volume without shell; d is the upper commercial diameter; ands Єis the experimental error.

As for the estimation of soil C stocks, the productivity of the NRs (195 m³ ha^−1^) at six years of age was used as a reference, allowing an estimate of WP contributions in the other treatments relative to this baseline. WPs have short- and medium-term potential for carbon sequestration. We assumed a basic wood density of 458 kg m^-3^ (Londero et al., 2015) and a C content of 446.1 g kg^-1^ of dry wood (Ribeiro et al., 2015) for the calculations.

### 2.5 Assessment of N_2_O and CH_4_ emissions from soil

To evaluate N_2_O and CH_4_ emissions, we used the closed static chamber method described by Mosier (1989). In each plot, a metal base (0.24 m² area) was inserted into the soil to a depth of 5 cm. A galvanized steel chamber [60 × 40 × 30 cm (L × W × H), 0.072 m³] was placed over a gutter fitted to the base, and water was added to the gutter to seal the system (Zanatta et al., 2010). Two internal fans, a septum connected to a three-way valve, and a digital skewer thermometer were installed in the upper part of the chamber to monitor internal temperature. The fans were powered by a battery and activated for 30 seconds immediately before sampling to homogenize the air inside the chamber. Air samples were collected using a 20 mL polypropylene syringe through the septum via the three-way valve.

The collections were performed at intervals of approximately 21 days between October 2016 and October 2017, totaling 18 collections. The samples were collected between 09:00 and 11:00 in the morning at 0, 20, 40, and 60 minutes after closing the chamber on the base. After collection, the samples were stored in exetainers and kept in a refrigerator at 4°C until analysis. The N_2_O and CH_4_ contents in the air samples were analyzed by gas chromatography in a GC-14 Greenhouse model equipped with an electron capture detector (ECD) and flame ionization detector (FID), using N_2_ as the carrier gas.

The N_2_O and CH_4_ fluxes were calculated based on the following equation:


$$f=\frac{\Delta Q}{\Delta t}\;\frac{PV}{RT}\;\frac{M}{A}$$

where *f* is the gas flux (μg m^−2^ h^−1^), ΔQ/Δt is the change in gas concentration (N_2_O or CH_4_), *P* is the atmospheric pressure inside the chamber (assumed to be 1 atm), *V* is the chamber volume (m³), *R* is the universal gas constant (0.08205 atm L mol^−1^ K^−1^), *T* is the temperature inside the chamber (K), *M* is the molar mass of the gas (g mol^−1^), and *A* is the chamber base area (m²).

The gas fluxes were measured between 9:00 and 11:00 a.m., a time interval considered the most representative of daily average GHG fluxes (Alves et al., 2012; Costa et al., 2008; Jantalia et al., 2008). Annual cumulative soil emissions of N_2_O and CH_4_ were calculated using the trapezoidal rule of integration based on the fluxes measured over one year.

The global warming potential (GWP), expressed in Mg C-CO_2_ equivalents, was estimated based on the annual emissions of C-CO_2_, N-N_2_O, and C-CH_4_ from the soil. Annual C-CO_2_ emissions were derived from changes in soil organic carbon (SOC) stocks for each treatment, using the NRs treatment as a reference. The rate of carbon retention in the soil was calculated as previously described. The GWP of the eucalyptus harvest residue management systems was determined by summing the annual emissions of the three greenhouse gases (GHGs), considering their respective global warming potentials relative to CO_2_ (N_2_O = 298, CH_4_ = 34, CO_2_ = 1), according to the following equation:


$$\begin{array}{c} GWP\;(kg\;CO_{2}eq\;ha^{-1}\;year^{-1}\\ =\left(N_{2}O\times 298\right)+\left(CH_{4}\times 34\right)+\;\left(\text{Δ}CO_{2}\right)+WPs\;C \end{array}$$

where GWP is the global warming potential; N_2_O and CH_4_ represent the annual emissions of N_2_O and CH_4_ from the soil in the respective harvest residue management systems, multiplied by their respective global warming potentials relative to CO_2_, considering a time horizon of 100 years (IPCC, 2006); ΔCO_2_ is the variation in soil C stocks in the other treatments in comparison with NRs, which was assumed to have remained similar to the stock before afforestation; WPs C is the amount of C stored in wood products.

### 2.6 Soil and weather parameters

Simultaneously with the gas flux assessments, we collected soil samples from the 0–10 cm layer to monitor ammonium (NH_4_^+^) and nitrate (NO_3_^-^) levels (Tedesco et al., 1995), and water-filled pore space (WPS) (Anderson et al., 2019). Particle density was determined from disturbed soil samples collected from the 0–10 cm layer at three random points in each treatment (Embrapa, 1997). Soil temperature was measured at a depth of 5 cm using a digital rod thermometer. Air temperature and rainfall data during the study period were obtained from an automatic meteorological station approximately 7 km from the experiment.

### 2.7 Statistical analysis

The variation of soil N_2_O and CH_4_ fluxes was expressed through the standard error of the mean. The CH_4_ and N_2_O flux data and soil parameters were correlated through Pearson’s correlation analysis. Linear regression analyses were used to verify the relationship between the input of C and N in the different eucalyptus harvest residue management systems and annual greenhouse gas (GHG) emissions. The GWP data were subjected to analysis of variance using the MIXED procedure (SAS, 2014) with the means compared by Tukey’s test at 10% significance.

## 3 Results

**Figure 1:**
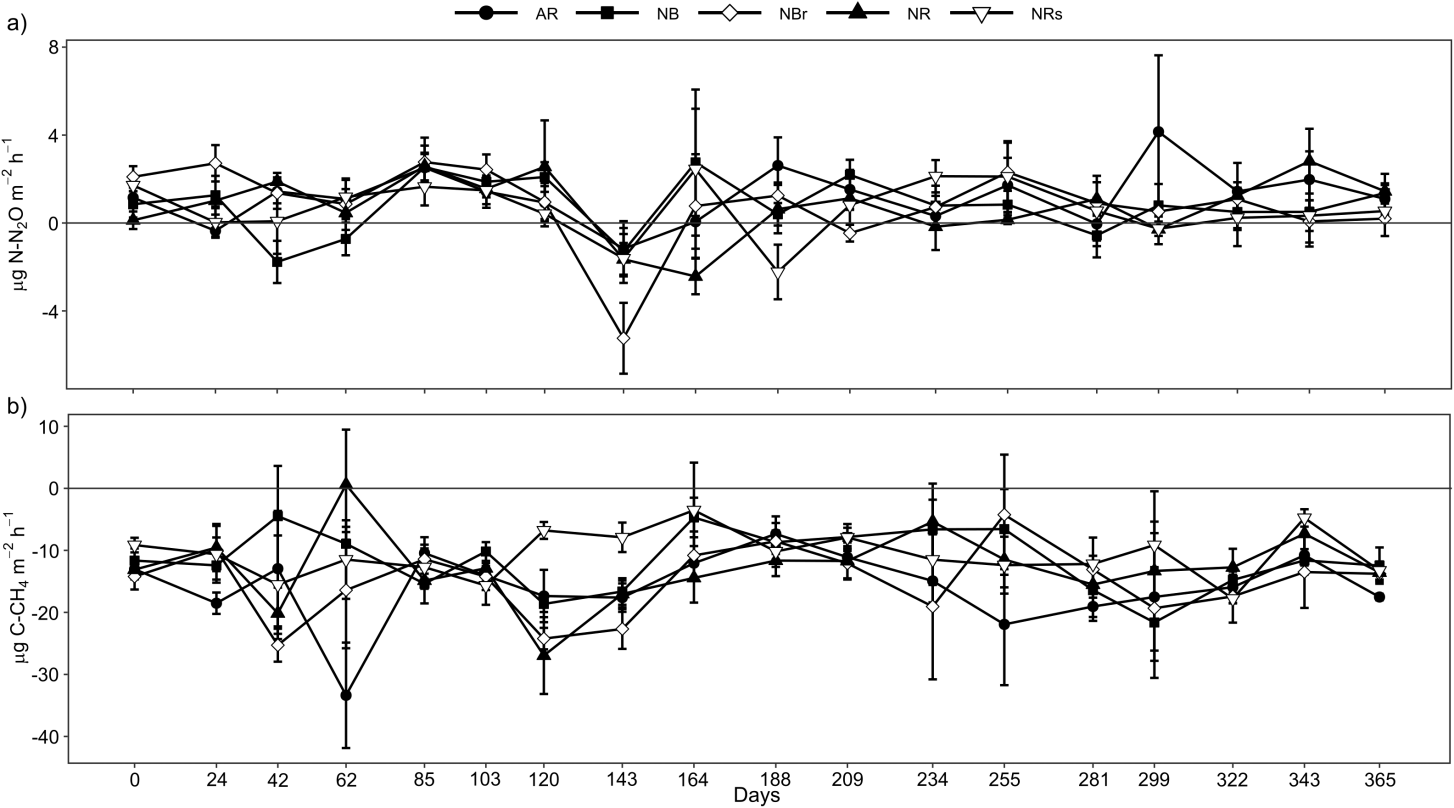
N_2_O **(a)** and CH_4_ **(b)** fluxes from a Quartzipsamment under different eucalyptus harvest residue management practices at six years of age, in Barra do Ribeiro, Brazil, over a one-year period.

Soil N_2_O fluxes ranged from -13.90 to 11.15 µg N-N_2_O m^-2^ h^-1^ in the different eucalyptus harvest management systems, characterizing a low intensity of fluxes in all treatments in this sandy soil (**Figure 1a**). Since no agricultural practices occurred during the sampling period, soil N_2_O fluxes remained practically constant without evident influence from eucalyptus harvest residues. N_2_O fluxes showed a low but significant correlation with soil NH_4_^+^ contents (**Table 1**).

**Table 1:** Pearson correlation between soil $NO_{3^{-}}$ and $CH_{4^{+}}$ fluxes, soil temperature (ST), air temperature (AT), NO₃⁻ and NH₄⁺ concentrations, and water-filled pore space (WFPS).

GHG	ST	AT	$NO_{3^{-}}$	$NH_{4^{+}}$	WPS
N_2_O	0,06^ns^	0,09^ns^	0,08^ns^	0,14^*^	0,09^ns^
CH_4_	-0,06^ns^	-0,09^ns^	-0,06^ns^	0,03^ns^	0,06^ns^

*ns: not significant; correlation coefficient (r) with p<0.05.

Soil CH_4_ flux ranged from -104.22 to 9.33 µg C ha^-1^ h^-1^, with a strong predominance of CH_4_ influx into the soil. As observed in N_2_O fluxes, there was no difference in CH_4_ influxes between eucalyptus harvest residue management systems (**Figure 1b**). However, there was a tendency for AR and NBr management to present higher CH_4_ oxidation rates compared to NRs in almost all evaluation periods.

To assess the impact of different management systems on soil C-CO_2_ retention, we used the system in which both harvest residues and litter were removed (NRs) as the reference. Based on this, the contributions of the other management systems to SOC retention were calculated relative to the NRs system. This approach — using the system with minimal organic input as a baseline — has been adopted by several authors in agricultural and forest systems (de Godoi et al., 2016; dos Santos et al., 2011; Dietz et al., 2024; Souza et al., 2023).

**Figure 2:**
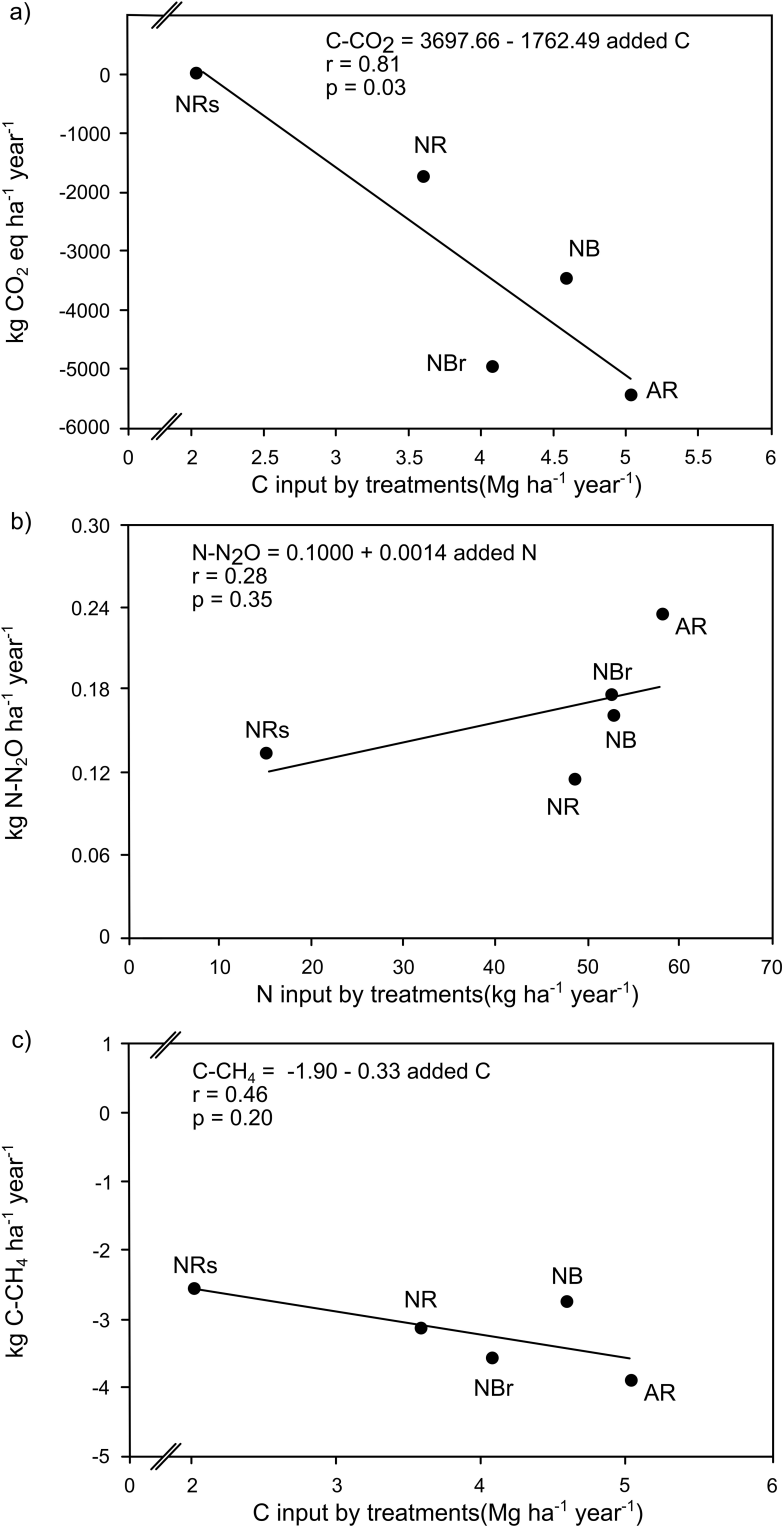
Annual rates of C-CO _2_ retention in the soil as a function of C input from eucalyptus harvest residues **(a)**; annual soil N-N _2_O emission as a function of mineral N input from eucalyptus harvest residues **(b)**; and annual C-CH₄ influx into the soil as a function of C input from eucalyptus harvest residues **(c)**.

A significant linear relationship was observed between the annual soil C-CO_2_ retention rates and the amount of carbon added through eucalyptus harvest residues and litter (r² = 0.81, p = 0.03) (**Figure 2a**). The highest retention rate (–5,540 kg C-CO_2_ ha^−1^ year^−1^) was recorded under the management system that retained both the previous crop’s residues and the current crop’s litter (AR). In contrast, the lowest rate (–1,752 kg C-CO_2_ ha^−1^ year^−1^) occurred under the system where only the current crop’s litter was retained, while the previous crop’s residues were removed (NR). Retention values similar to those in the AR system were observed in the two systems that maintained either bark or branches (NB and NBr).

Annual soil N_2_O emissions ranged from 0.11 to 0.23 kg N-N_2_O ha^−1^ year^−1^ and did not show a direct relationship with the amount of N added by eucalyptus harvest residues and litter (r² = 0.28; p = 0.35) (**Figure 2b**). Regarding methane during the one-year evaluation period, the soil showed an annual uptake of -2.56 to -3.91 kg C-CH_4_ ha^−1^ year^−1^, with a weak direct correlation with the amount of C added by harvest residues and litter (**Figure 2c**).

**Figure 3:**
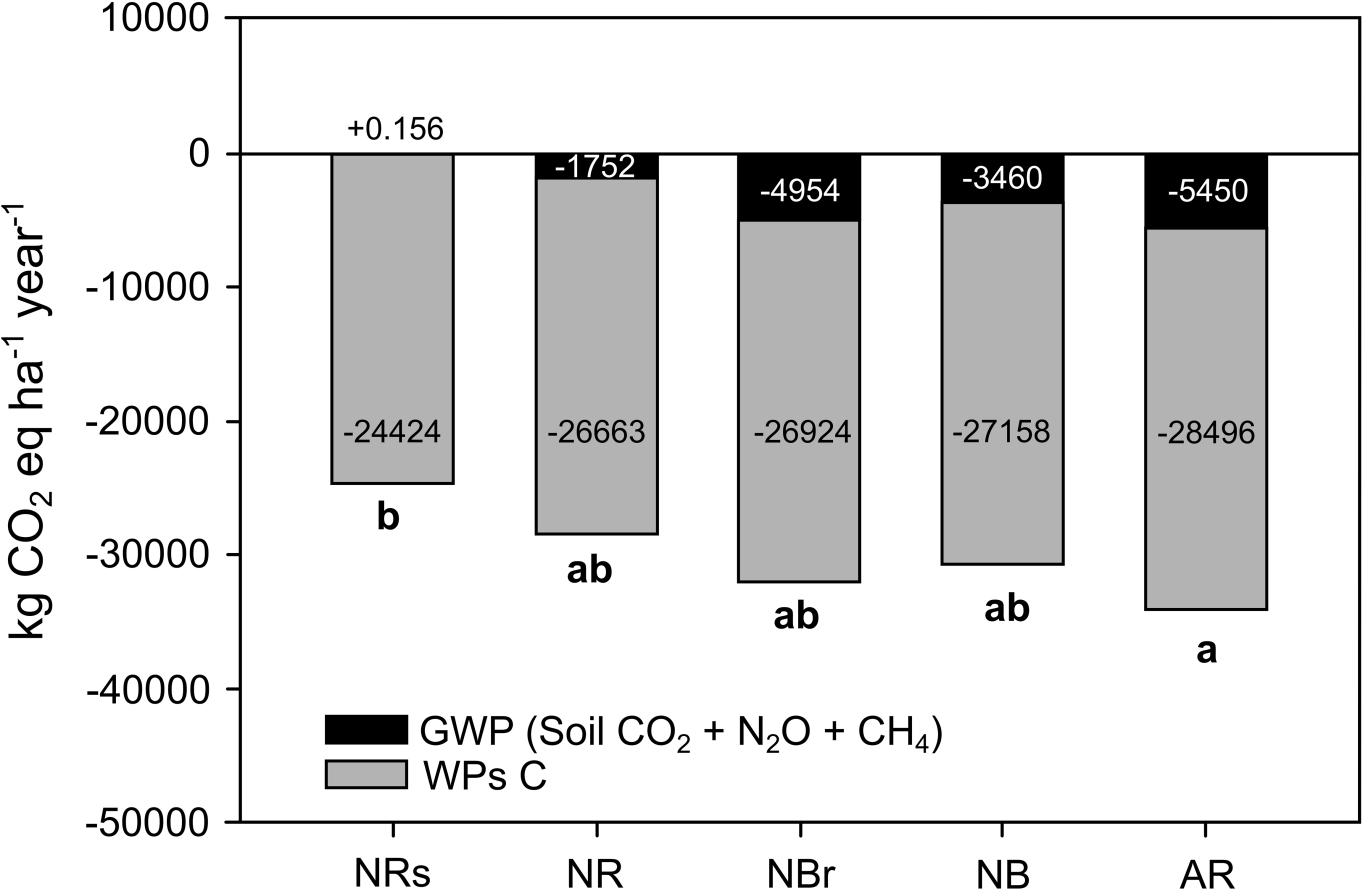
Cumulative emissions and global warming potential of the soil under eucalyptus harvest residue management. Means followed by the same letter do not differ from each other according to Tukey’s test at 10%.

All treatments demonstrated potential for mitigating global warming. The GWP values ranged from –24,424 to –33,946 kg CO_2_eq ha^−1^ year^−1^ (**Figure 3**). The main contributors to this atmospheric carbon sequestration were the carbon added to the soil and the carbon stored in wood products, which offer medium- and long-term sequestration potential.

The management system that retained eucalyptus harvest residues (AR) showed the lowest GWP (–33,946 kg CO_2_eq ha^−1^ year^−1^), representing a significant reduction in greenhouse gas emissions compared to the NRs system (p< 0.10). AR was followed by NBr (–31,879 kg CO_2_eq ha^−1^ year^−1^), NB (–30,619 kg CO_2_eq ha^−1^ year^−1^), and NRs (–28,416 kg CO_2_eq ha^−1^ year^−1^), with no significant differences among these three treatments.

In the reference system (NRs), nearly all the GWP value was attributable to carbon stored in wood. In contrast, wood carbon contributed approximately 85% of the total GWP on average in the systems with partial or complete retention of eucalyptus residues. The remaining share was associated with the influence of harvest residue management on soil organic carbon retention.

## 4 Discussion

The N_2_O fluxes we observed are consistent with recent studies in eucalyptus areas in tropical Brazilian soils. Cuer et al. (2018) found values below 10 μg N-N_2_O m^−2^ h^−1^. Silveira et al. (2022) observed that eucalyptus forests emitted up to 5.5 μg N-N_2_O m^−2^ h^−1^ during the rainy spring, but acted as a N_2_O sink during the dry winter periods.

In general, eucalyptus plantations established in sandy soils have low nitrogen availability and, consequently, low N_2_O emissions (Livesley et al., 2009). In addition to the low levels of N-NH_4_^+^ and N-NO_3_^-^ in the soil, we also observed low WPS values, which may have contributed to the low N_2_O emission (**Supplementary Figure S1**). The WPS values were below 25%, an unfavorable condition for N_2_O production by denitrification, which occurs at WPS values above 60% (Bateman and Baggs, 2005). In sandy soils, such as the one examined in this study, oxygen diffusion rates are higher than in clay soils, avoiding anaerobic conditions for a prolonged period, a key requirement for denitrification (Rochette et al., 2008). The lack of relationship between N_2_O fluxes and soil N-NO_3_^-^ levels reinforces this hypothesis. On the other hand, the correlation with N-NH_4_^+^ contents suggests that the nitrification process may have contributed significantly to N_2_O fluxes. During nitrification, a process favored under aerobic conditions, intermediate compounds may lead to non-obligatory N_2_O production (Zhang et al., 2025).

The impact of plant residues on N_2_O emissions depends on the composition of these residues, especially their C/N ratio (Li et al., 2016). In general, emissions are negatively correlated with the C/N ratio, meaning that the presence of residues with a high C/N ratio promotes N immobilization, reducing net mineralization and N_2_O production (Pilegaard et al., 2006). In this context, our results support the idea that the input of forest residues with a C/N ratio greater than 30 has a low contribution to N_2_O emissions (Fest et al., 2015) due to the rapid immobilization and limited availability of N for nitrification and denitrification processes (Livesley et al., 2009). The C/N ratio values in our treatments ranged from 111.7 to 162.7, accompanied by high lignin/N ratios, 58.1 to 69.1 (São José et al., 2023), which favor microbial nitrogen immobilization. Additionally, the low-organic-matter sandy soil was fertilized only during forest establishment (São José et al., 2020), contributing to low nitrogen availability and, consequently, low N_2_O emissions.

However, since we did not observe differences between treatments, our results differ from other studies involving agricultural residue management (Gonzaga et al., 2018; Maris et al., 2018; Vasconcelos et al., 2018; Reeves et al., 2024). These differences are possibly related to the period between the application of residues and the collection of gas samples. In those studies, the highest N_2_O emissions occurred immediately after the addition of plant residues, with a reduction after a few months and remaining constant over time. In our study, the evaluations only occurred six years after the experiment started. Thus, we believed that N_2_O emissions in the management of eucalyptus harvest residues were already reduced and stabilized, and possibly the highest emissions must have occurred at the initial time of the experiment installation.

The negative CH_4_ fluxes we observed are consistent with other studies conducted in eucalyptus areas (Fest et al., 2017; Fialho et al., 2018; Silva et al., 2024). In our case, the influxes were possibly favored by the sandy texture of the soil, which, regardless of the management adopted, allowed greater oxygen diffusion and, consequently, created conditions for CH_4_ oxidation. This assumption is supported by the findings of Livesley et al. (2011) and Grover et al. (2012), who observed similar CH_4_ dynamics in low-nutrient sandy soils of northern Australian Eucalypt savanna woodlands.

Methane influxes may also be related to low WPS values that favor CH_4_ oxidation (Liu et al., 2019). In such a condition, the improved soil porosity and gas diffusivity facilitate the transport of CH_4_ to methanotrophic bacteria that oxidize CH_4_ to CO_2_. CH_4_ influxes are usually inversely related to soil moisture (Fest et al., 2017; Liu et al., 2019). Despite this, in the present study, no relationship was observed between CH_4_ influxes and WPS, probably due to the reduced water retention capacity of the sandy soil. CH_4_ influx is enhanced in sandy soils, where rapid drainage occurs, preventing the maintenance of high soil water contents for prolonged periods that could determine soil reduction conditions (Walkiewicz et al., 2025). Our results were similar to recent studies on agricultural residue management (Wegner et al., 2018; Langeroodi et al., 2019) and in harvesting and soil preparation operations in eucalyptus areas (Fialho et al., 2018). As in our study, these authors also attributed the low effect of plant residues on CH_4_ fluxes to the small variation in soil moisture between residue managements.

Removing eucalyptus harvest residues has been widely considered a management that reduces C-CO_2_ retention in the soil (Rocha et al., 2018). This practice is more relevant in soils with lower clay contents, which have a lower capacity for physical protection of soil organic matter (Dieckow et al., 2009), causing reductions in soil C compared to the maintenance of eucalyptus harvest residues (Oliveira et al., 2018). The results obtained in this study corroborate these considerations. In addition, the capacity of the soil to function as a CO_2_ sink depends on the biomass input (Conceição et al., 2013). This dependence is evidenced by the correlation between C-CO_2_ retention rates in the soil and the amount of C contributed by eucalyptus harvest residues and litter (**Figure 2a**), reinforcing the importance of maintaining eucalyptus harvest residues to promote C additions, mainly in sandy soils in tropical regions (Epron et al., 2015).

On the other hand, annual N_2_O emissions were not correlated with the amount of N added by plant residues. Much of the N present in the residues may have already been released in the first months of implementation of the experiment, as observed by Rocha et al. (2016). The authors evaluated the decomposition and release of nutrients in different management systems of eucalyptus harvest residues, observing that the management system with the maintenance of all residues released approximately 130 kg ha^-1^ of N after 300 days from the beginning of the experiment. In addition, the high C/N ratio of the bark and branches of the residues (110 and 316, respectively) and the low N content of the litter could be causing the immobilization of N by the soil microbial population.

Studies evaluating annual N_2_O emissions in reforestation areas in subtropical regions have shown variable results. The annual N_2_O emissions in our study were lower than those observed in areas with *Acacia mearsii* (0.24 ± 1.25 kg N ha^-1^ year^-1^) (de Godoi et al., 2016), *Acacia auriculiformis* (2.3 ± 3.1 kg N ha^-1^ year^-1^), and *Eucalyptus urophylla* (1.9 ± 2.1 kg N ha^-1^ year^-1^) (Zhang et al., 2014). However, our results were quite similar to those obtained by van Delden et al. (2018), who observed annual N_2_O emissions ranging from 0.08 to 0.09 kg N ha^-1^ year^-1^ in eucalyptus areas grown in subtropical sandy soils in Australia. Our results demonstrate the low potential for N_2_O emissions in the different management of eucalyptus harvest residues in this Brazilian sandy soil.

Despite the low linear relationship between eucalyptus harvest residue input and CH_4_ influxes, we observed a trend of lower influx in the NRs and NR systems, which may be related to lower soil quality compared to the AR system (São José et al., 2022). The loss of soil quality results in a lower capacity to oxidize CH_4_ (Bayer et al., 2013). Wu et al. (2019) obtained similar results. The authors observed that litter removal reduced the CH_4_ oxidation capacity by approximately 30% compared to areas that maintained litter in coniferous forests in the Chinese subtropics. This reduction was attributed to the lower abundance of methanotrophic microorganisms due to the poor availability of low-molecular-weight organic compounds caused by litter removal.

Carbon sequestration is considered one of the main factors controlling GWP in agricultural systems (Schönbach et al., 2012), which was confirmed by our results. Furthermore, our results demonstrate that N_2_O and CH_4_ fluxes made a negligible contribution to the final GWP result, as typically observed in forests (Saggar et al., 2008; Walkiewicz et al., 2025). Our results are similar to those obtained by Zhang et al. (2015), who observed that the contributions of N_2_O and CH_4_ to GWP were less than 3% in forest areas in the subtropical region of China. In our study, however, the contribution of these gases was even lower, not reaching 1% (**Supplementary Table S2**).

Few studies have related the effects of forest management impacts with GWP, and there is no standardization in the calculations of this variable, which makes it difficult to compare results. Martins et al. (2015) observed a 76% reduction in GWP in *Eucalyptus saligna* areas in Australia compared to pasture areas. However, these authors did not consider the soil C retention rate and included only CH_4_, N_2_O, and CO_2_ emissions. Wang et al. (2022) also adopted this approach, excluding soil carbon retention rates and considering only CH_4_, N_2_O, and CO_2_ emissions in their calculations. They were among the few, if not the only, researchers to evaluate the effect of harvest residue management on GHG emissions in forest ecosystems. However, their study focused on *Cunninghamia lanceolata* growing in a soil type markedly different from ours, with approximately eight times higher organic matter content. de Godoi et al. (2016), studying *Acacia mearnsii* areas in the Brazilian subtropics, found that wood contributed approximately 70% of the GWP value, while soil carbon retention accounted for about 30%.

Estimating the GWP of local agricultural and forest systems is essential for obtaining accurate and context-specific assessments of environmental impacts. To the best of our knowledge, this is the first study to evaluate the effects of eucalyptus harvest residue management on the GHG balance in a Brazilian subtropical sandy soil. In our research, the management practice that retained all eucalyptus harvest residues and litter (AR) showed significantly lower soil-associated GWP values, indicating a greater potential for carbon sequestration compared to other management strategies. These results demonstrate that maintaining eucalyptus harvest residues, besides promoting an increase in forest productivity, represents an alternative for mitigating GHG emissions in subtropical sandy soils, both due to the potential for CH_4_ oxidation and the storage of soil organic carbon.

## Data Availability

The raw data supporting the conclusions of this article will be made available by the authors, without undue reservation.
