# Supplementary material for: Reducing greenhouse gas emissions via harvest residue management in eucalyptus afforestation on Brazilian sandy soils
Source: Front Plant Sci. 2025 Aug 11;16:1633436. doi: 10.3389/fpls.2025.1633436 (PMC12375687; doi:10.3389/fpls.2025.1633436)
Supplement: Supplementary file 1 [file DataSheet1.docx]

Supplementary Material

**Supplementary Table 1.** Soil chemical and physical attributes prior to the implementation of the experiment.

| Deph | O.M | pH (H_2_O) | Al | H + Al | CEC  efet. | | CEC pH 7 | | Ca | | | Mg | P | | K |
| --- | --- | --- | --- | --- | --- | --- | --- | --- | --- | --- | --- | --- | --- | --- | --- |
| (cm) | dag kg^-1^ |  | -------------------------cmol_c_ dm^-3^-------------------- | | | | | | | | | | ---- mg dm^-3^--- | | |
| 0-20 | 0.6 | 4.9 | 0.7 | 2.1 | 1.5 | | | 2.9 | | 0.6 | | 0.2 | 8.4 | | 16.0 |
| 20-40 | 0.5 | 5.1 | 0.4 | 2.4 | 1.4 | | | 3.4 | | 0.8 | | 0.2 | 13.2 | | 14.2 |
| 40-60 | 0.4 | 5.1 | 0.8 | 2.0 | 1.6 | | | 2.8 | | 0.6 | | 0.1 | 10.2 | | 13.0 |
| 60-80 | 0.3 | 5.0 | 0.8 | 2.3 | 1.5 | | | 3.0 | | 0.6 | | 0.1 | 9.5 | | 12.0 |
| 80-100 | 0.3 | 5.1 | 0.8 | 2.6 | 1.4 | | | 3.2 | | 0.5 | | 0.1 | 9.3 | | 14.6 |
| Deph | m | BS | Sandy coarse | | Sandy fine | | | | | Silt | | | Clay | | |
| (cm) | --------------------------------------------------%------------------------------------------------- | | | | | | | | | | | | | | |
| 0-20 | 47.1 | 27.7 | 93.1 | | | 3.6 | | | | | 0.0 | | | 3.3 | |
| 20-40 | 36.0 | 27.0 | 92.8 | | | 5.8 | | | | | 0.0 | | | 1.4 | |
| 40-60 | 52.2 | 26.3 | 91.9 | | | 6.3 | | | | | 0.0 | | | 1.7 | |
| 60-80 | 56.5 | 22.8 | 92.8 | | | 5.0 | | | | | 0.0 | | | 2.2 | |
| 80-100 | 57.6 | 19.3 | 92.2 | | | 5.5 | | | | | 0.0 | | | 2.3 | |

P and K: Mehlich^-1^ Extractor; Al, Ca and Mg: KCl 1 mol l^-1^ Extractor; H + Al: Ca(OAc)2 0.5 mol l^-1^ pH 7.0

Extractor; O.M. = Organic matter; CEC = Cation Exchange capacity; m = Aluminium saturation percentage;

BS = Base saturation percentage.

**Supplementary Table 2.** Relative importance of each greenhouse gas (GHG) to global warming potential (GWP)

| **Treatament** | **GHG** | **GWP (kg CO₂ eq ha⁻¹ ano⁻¹)** | **Net GWP** | **Contribution(%)** |
| --- | --- | --- | --- | --- |
| NRs | N₂O | 0.2181 | -3.4449 | -6.33% |
|  | CH₄ | -3.6630 |  | 106.33% |
|  | CO₂ | 0 |  | 0.00% |
|  |  |  |  |  |
| NR | N₂O | 0.2240 | -1756.4444 | -0.01% |
|  | CH₄ | -4.3932 |  | 0.25% |
|  | CO₂ | -1752.2752 |  | 99.76% |
|  |  |  |  |  |
| NBr | N₂O | 0.2904 | -4959.5796 | -0.01% |
|  | CH₄ | -5.1396 |  | 0.10% |
|  | CO₂ | -4954.7304 |  | 99.90% |
|  |  |  |  |  |
| NB | N₂O | 0.2654 | -3463.9612 | -0.01% |
|  | CH₄ | -3.9495 |  | 0.11% |
|  | CO₂ | -3460.2771 |  | 99.89% |
|  |  |  |  |  |
| AR | N₂O | 0.4057 | -5455.4035 | -0.01% |
|  | CH₄ | -5.4967 |  | 0.10% |
|  | CO₂ | -5450.3126 |  | 99.90% |


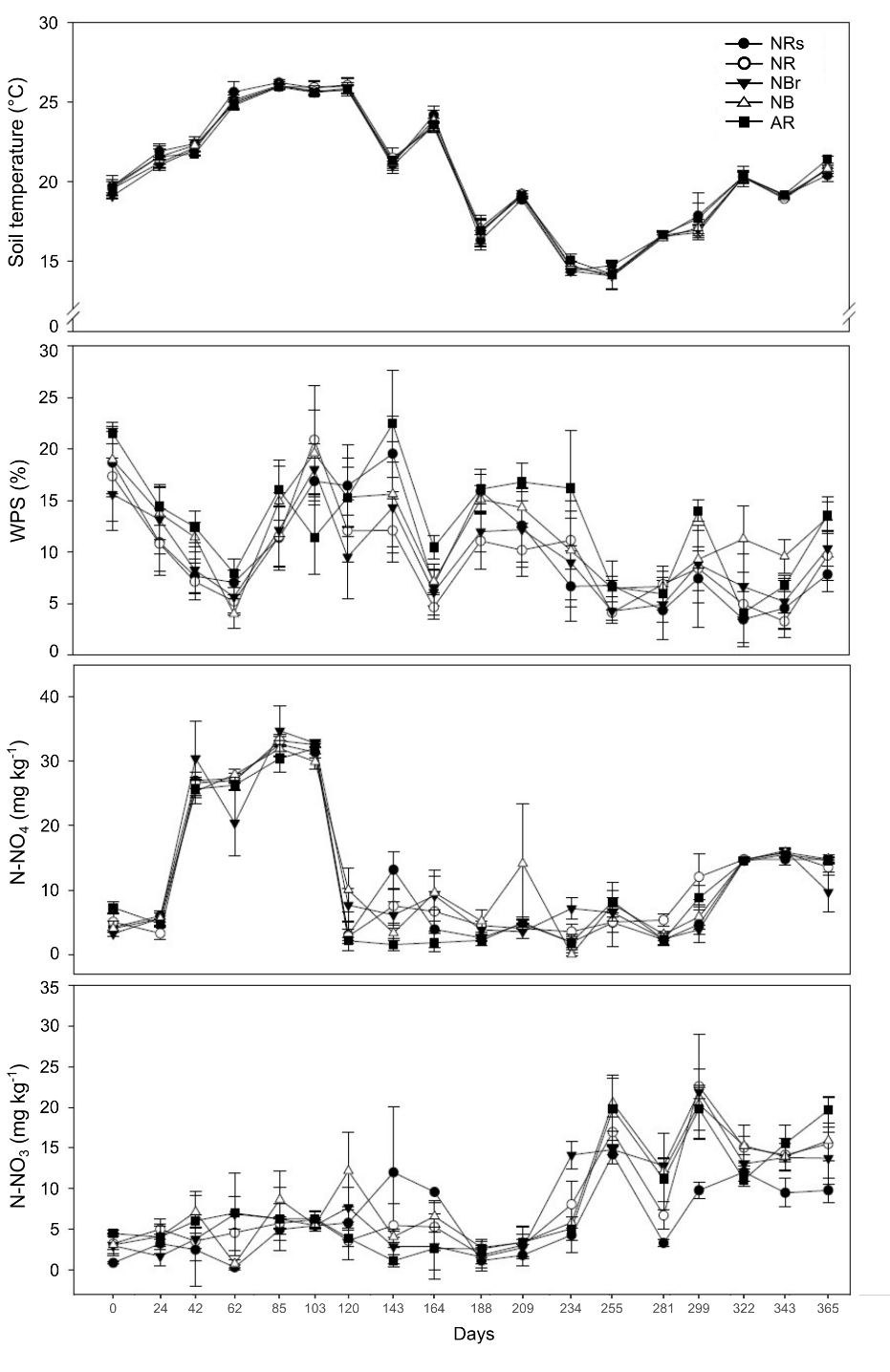


**Supplementary Figure 1.** Soil temperature (a), water-filled pore space (WPS) (b), N-NH4 content (c), N-NO3 content (d) in the 0 to 10 cm soil layer in different management of eucalyptus harvest residues in Quartzarenic Neosol at six years of age. Barra do Ribeiro, October/2016 to October/2017. NRs-Removal of all residues from the previous crop (bark, branches, and leaves) and litter from the new planting through the use of shade cloth; NR-Removal of all harvest residues (bark, branches, and leaves) + litter from the new planting; Nbr-Maintenance of bark and leaves + litter from the new planting; NB-Maintenance of branches and leaves + litter from the new planting; AR-Maintenance of all residues (bark, branches, and leaves from the previous crop) + litter from the new planting. The vertical bars represent the standard error of the mean.
